# Abstracts

**Published:** 1893-04

**Authors:** 


### Abstracts.

SYMPTOMS OF BRAIN INJURIES.— *Compression.*—(1.) Total insensibility. (2.) Respiration, stertorous and puffing. (3.) Pulse slow, full and labored. (4.) Special senses paralyzed. (5.) Pupils dilated or one dilated and the other contracted. (6.) Stomach inactive. Sphincters may be paralyzed. Bowels torpid. Bladder paralyzed. Retention appears gradually, and grows worse and worse.

*Concussion.*—(1.) Insensibility from which the patient can be partly aroused. (2.) Respiration feeble. (3.) Pulse weak and irregular. (4.) Special senses dulled. (5.) Pupils variable. Sensitive to light. Bowels relaxed. Sphincters not relaxed. Bladder can expel water. Comes instantaneously and passes off gradually.—*St. Louis Clinique.*

ADVERTISING BY PHYSICIANS.—The editor of the Cincinnati *Lancet-Clinic* discusses the pros and cons of this question at length, and concludes his consideration of the subject by the following advice:

"Leaving the newspaper out of consideration, a physician has two legitimate channels for advertising—first, the medical journal; and second, the reputation of knowing and attending to his business. In other words, ability to be a scientific physician is of more pecuniary value to a man than all the newspaper advertising he can possibly receive. 'The best advertisement for a physician is the confidence and respect of his brother-physicians.' This is a truism of great value and of far-reaching import. If all physicians appreciated the value of this confidence and respect, there would be fewer men anxious to appear in the public press."—*Medical Age.*

POISONING BY ANTISEPTICS.—Prof. Albert, at a recent meeting of the Vienna Medical Society, suggested the possibility of surgeons suffering from chronic mercurial poisoning from the constant use of bichloride in surgical operations. Prof. Albert says that he for some time suffered from dyspepsia, for which he could assign no cause. An examination of his urine showed iodide of mercury in comparatively large quantities, considering the mode of its absorption. He has also lost three healthy teeth and noticed a softening of the finger-nails.—*Courier of Medicine.*

